# Supplementary material for: Comprehensive Annotation and Functional Exploration of MicroRNAs in Lettuce
Source: Front Plant Sci. 2021 Dec 24;12:781836. doi: 10.3389/fpls.2021.781836 (PMC8739914; doi:10.3389/fpls.2021.781836)
Supplement: Supplementary file 3 [file Data_Sheet_9.ZIP › GSM2230753.plot/Lsa-miRN1649_Lsat_1_v5_gn_4_85420.1_3038_TPlot.pdf]

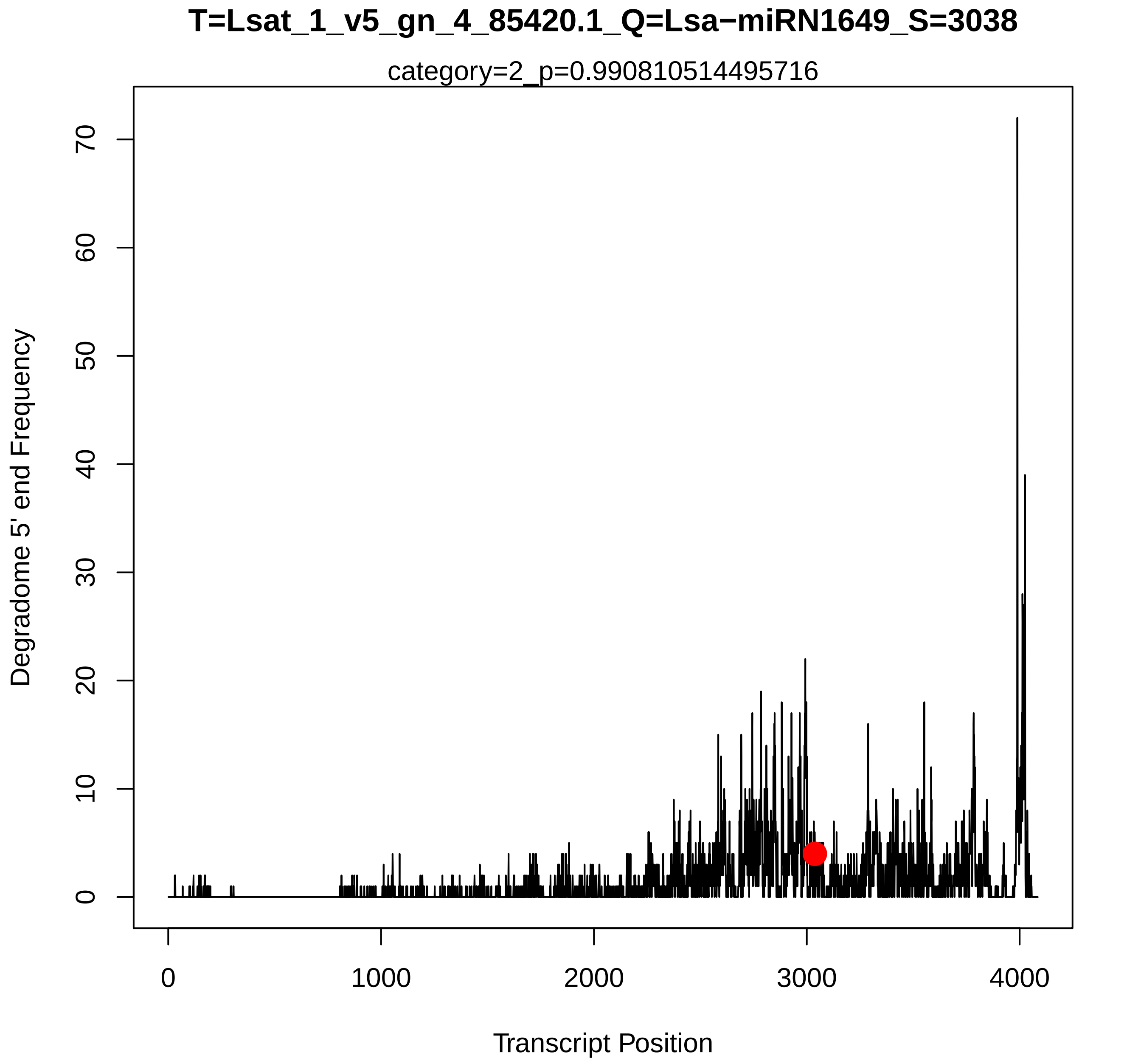
T=Lsat_1_v5_gn_4_85420.1_Q=Lsa−miRN1649_S=3038
category=2_p=0.990810514495716
Degradome 5' end Frequency
0
10
20
30
40
50
60
70
0
1000
2000
3000
4000
Transcript Position
